# Qualitative and Quantitative Analysis of *Eclipta prostrata* L. by LC/MS

**DOI:** 10.1155/2015/980890

**Published:** 2015-01-08

**Authors:** Lifeng Han, Erwei Liu, Agyemang Kojo, Jing Zhao, Wei Li, Yi Zhang, Tao Wang, Xiumei Gao

**Affiliations:** ^1^Tianjin State Key Laboratory of Modern Chinese Medicine, Tianjin University of Traditional Chinese Medicine, 312 Anshanxi Road, Nankai District, Tianjin 300193, China; ^2^Tianjin Key Laboratory of TCM Chemistry and Analysis, Tianjin University of Traditional Chinese Medicine, 312 Anshanxi Road, Tianjin 300193, China

## Abstract

*Eclipta prostrata* L. is one of the Chinese medicinal tonics which are usually used for treating loose teeth, dizziness, tinnitus, hemoptysis, hematuria, and uterine bleeding. However, quality control of this herbal medicine has been not satisfactory. This study reported its qualitative and quantitative analyses based on LC/MS method. UHPLC-DAD-Q-TOF-MS fingerprinting and MS fragmentation cleavage pathway were investigated for qualitative analysis. Furthermore, a method for simultaneous quantitative determination of nine compounds, luteolin 7-*O*-*β*-D-glucopyranoside, ecliptasaponin C, luteolin, eclalbasaponin IV, apigenin, ecliptasaponin A, echinocystic acid 28-*O*-*β*-D-glucopyranoside, echinocystic acid, and 3-oxo-16*α*-hydroxy-olean-12-en-28-oic acid in *E. prostrata*, was established. The method was validated for samples of *E. prostrata* from different habitats. The results showed good linear correlation, precision, accuracy, and repeatability that could be used for contents determination of the nine compounds in *E. prostrata* from different habitats.

## 1. Introduction

*Eclipta prostrata* L. (Compositae) is one of the Chinese medicinal tonics, widely distributed in the tropical and subtropical regions of the world. It has been used for the treatment of loose teeth, dizziness, tinnitus, hemoptysis, hematuria, and uterine bleeding [1]. Modern pharmacological research has confirmed its biological effects such as in antiosteoporosis [2], anti-inflammatory [3], antihyperlipemia [4, 5], and antitumor [6] activities.

Literature on its phytochemical constituents and our previous study showed that it contained triterpenoid saponins, flavonoids, thiophenes, and steroids [6–10]. Several analytical methods including HPLC, UPLC, LC/MS, and GC-MS have been used for quality control analyses of* E. prostrata *[11–15]; however, the standards which those methods used were finite (one or two flavonoids which could be obtained on the market). Furthermore, analytical time of the reported methods was relatively long in order to obtain ideal resolution. Therefore, we intended to establish a method which is based on our previous phytochemical study to determine main constituents of* E. prostrata.* Although there was a report on qualitative analysis of* E. prostrata* through UHPLC-Q-TOF/MS [12], the results lacked to deduce fragmentation pathway since they did not contain MS/MS data.

In this paper, we demonstrated how modern analytical methods could be used for quality control on natural product medicine. We initially analyzed a UHPLC-DAD-Q-TOF-MS fingerprint for rapid profiling of chemical constituents, and eighteen compounds in the extract of* E. prostrata* were identified or tentatively characterized. A rapid LC-QQQ-MS method was later validated for simultaneous determination of nine major compounds in* E. prostrata*. The results showed good linear correlation, precision, accuracy, and repeatability that could be used for quality control analysis of* E. prostrata* from different habitats.

## 2. Experimental

### 2.1. Reference Substances, Reagents, and Plant Materials

**Figure 1:**
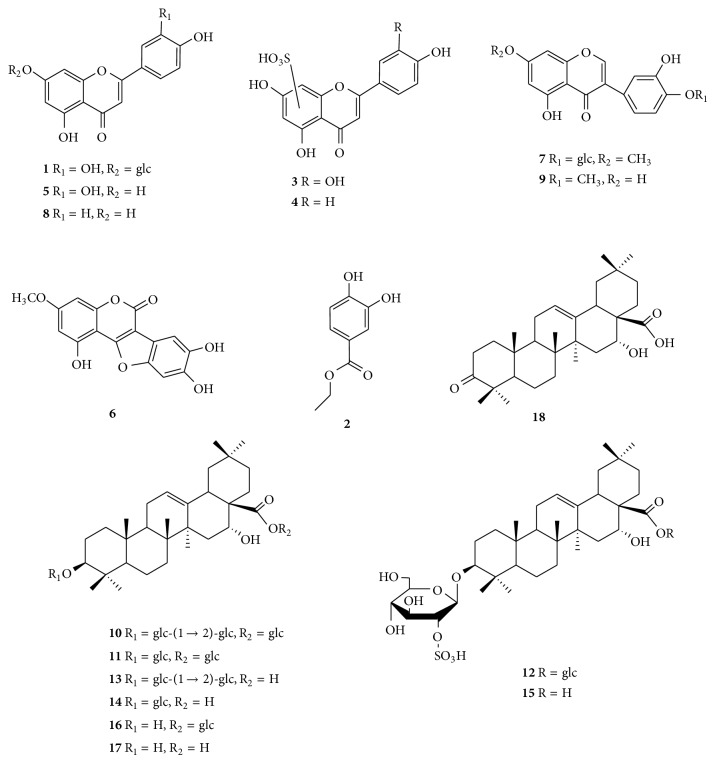
The structures of compounds** 1**–**18 **identified from* Eclipta prostrata *L.

The references (**1**,** 2**,** 5**–**9**,** 11**,** 13**,** 14**, and** 16**–**18)** (Figure 1) were isolated from the aerial part of* E. prostrata*, and the structures elucidated based on ^1^H-NMR and ^13^C-NMR spectral analyses as previously reported by [10]. The purities of these reference compounds were determined to be above 98% by normalization of the peak areas detected by HPLC-ELSD (Alltech Grace Evaporative Light Scattering Detector 3300 with the following acquisition parameters: temp: 40°C; gas flow: 1.8 L/min; gain: 10).

HPLC grade acetonitrile and methanol were acquired from Fisher Chemicals (Pittsburg, USA). Formic acid (HPLC grade) was obtained from Tedia, USA. The other chemicals and reagents used were of analytical grade and purchased from Tianjin Concord Technology Company (Tianjin, China). Purified water was obtained using a Milli-Q system (Millipore, USA).

Thirteen dried samples (S1–S13) from the aerial part of* E. prostrata* were collected from different habitats and identified by Professor Lijuan Zhang (College of Traditional Chinese Medicine, Tianjin University of Traditional Chinese Medicine, China). Voucher specimens (20120903) deposited in our laboratory.

### 2.2. Preparation of the Reference and Sample Solutions

A concentration of 1 mg/mL of the reference compounds** 1**,** 2**,** 5**–**9**,** 11**,** 13**,** 14**, and** 16**–**18** was prepared using 50% methanol (v/v) for UHPLC-Q-TOF-MS analysis.

For LC-QQQ-MS analysis, nine of the reference compounds were accurately weighed, put into 10 mL volumetric flasks separately, and dissolved in 50% methanol (v/v) to make reference stock solution 1. Their respective concentrations were as follows: luteolin 7-*O*-*β*-D-glucoside (18.15 mg/mL), ecliptasaponin C (47.05 mg/mL), luteolin (4.64 mg/mL), eclalbasaponin IV (11.85 mg/mL), apigenin (1.56 mg/mL), ecliptasaponin A (21.50 mg/mL), echinocystic acid 28-*O*-*β*-D-glucoside (2.60 mg/mL), echinocystic acid (8.45 mg/mL), and 3-oxo-16*α*-hydroxy-olean-12-en-28-oic acid (6.42 mg/mL). 1 mL of the stock solution 1 was then further diluted with 50% methanol (v/v) to 10 mL to obtain reference stock solution 2. All the solutions were stored at 4°C and brought to room temperature before use. Calibrated reference working solutions were freshly prepared by appropriate dilution of the mixed stock solution 2, giving final concentration in the range of 0.028–20.17 *μ*g/mL for** 1**, 0.070–52.28 *μ*g/mL for** 11**, 0.012–8.59 *μ*g/mL for** 5**, 0.060–43.89 *μ*g/mL for** 13**, 0.008–5.78 *μ*g/mL for** 8**, 0.033–23.89 *μ*g/mL for** 14**, 0.013–9.63 *μ*g/mL for** 16**, 0.013–9.39 *μ*g/mL for** 17**, and 0.010–7.13 *μ*g/mL for** 18**.

The aerial part of* E. prostrata* was crushed into powder and 1.00 g weighed into a 50 mL flask with 25 mL 50% methanol (v/v). It was extracted in an ultrasonic bath at room temperature for 30 min. The extract was filtered. 1 mL of the filtrate was obtained and diluted to the mark in a 10 mL volumetric flask. It was then centrifuged at 13171 ×g for 10 min for LC/MS analysis.

### 2.3. UHPLC-DAD-Q-TOF-MS Fingerprint

UHPLC-DAD-Q-TOF-MS fingerprint analysis was performed on an Agilent 1290 UHPLC consisting of a binary pump, a diode-array detector, an autosampler, and a column thermostat connected to an Agilent 6520 Q-TOF spectrometry system via an ESI interface (Agilent Corp., Santa Clara, CA, USA).

The peaks were separated on an ACQUITY UPLC HSS T_3_ column (100 mm × 2.1 mm, 1.8 *μ*m, Waters, USA) using a mobile phase consisting of acetonitrile (A) and water (containing 0.1% HCOOH; B). The following gradient elution system was used: 0–15 min, 10–30% A; 15–28 min, 30% A; 28–30 min, 30–40% A; 30–38 min, 40% A; 38–45 min, 40–100% A; 45–50 min, 100–100% A; 50–50.5 min, 10% A; 50.5–60 min, 10% A. An injection volume of 5 *μ*L and a flow rate of 0.3 mL/min were used, with the column temperature set at 30°C.

Full-scan analyses in negative ionization modes were conducted and the spectra were recorded in the range of* m/z* 100–1700. Liquid nitrogen was used as the nebulizer and drying gas. High purity nitrogen was used as the collision gas. The major parameters used were as follows: drying gas with a flow rate of 8.0 L/min, drying gas temperature 350°C, nebulizer 30 psig, capillary voltage 3500 V, fragmentor voltage 175 V, and collision energy 35, 70 V.

### 2.4. Simultaneous Quantification System

The mass spectrometer was an Agilent 6430 triple Quad MS (QQQ) coupled with Agilent 1200 HPLC, consisting of a binary pump, an online vacuum degasser, an autosampler, and a column oven, through an electrospray ionization (ESI) source (Agilent Corp., Santa Clara, CA, USA). All separations were carried out on an Agilent eclipse XDB-C18 column (150 mm × 4.6 mm, 5.0 *μ*m, Agilent, USA) and the column temperature was set at 35°C. The mobile phase was composed of acetonitrile (A)-water (containing 0.1% HCOOH; B) at a gradient elution of 0–5 min, 30–100% A; 5–10 min, 100% A; 10-11 min, 100–30% A; 11–20 min, 30% A, with a flow rate of 0.5 mL/min, and 1 *μ*L injection volume.

The ESI source parameter of drying gas was set at the flow rate of 8.0 L/min, temperature of 350°C, 45 psig nebulizer, and 4000 V capillary voltage. The MS/MS analysis was conducted on multiple reactions monitoring (MRM) mode; the MRM parameters were shown in Table 1.

### 2.5. Method Validation for Simultaneous Quantification

Seven serial working solutions were prepared as described above and injected into the LC/MS system. Calibration curves were plotted based on linear regression analysis of the integrated peak areas (*Y*) versus concentrations (*X*, *μ*g/mL). Each solution was tested in triplicate, with limits of detection (LOD) and quantification (LOQ) for each analyte defined at signal-to-noise ratios (*S*/*N*) of 3 and 10, respectively. Intraday and interday precision for each analyte at a specific concentration were performed by six replicates on the same day (intraday) and on three consecutive days (interday). Recovery tests were performed by spiking reference standards into appropriately weighed sample 9. Six different samples were spiked with the reference standards, extracted, and prepared as described above. Three replicates were performed for each analysis. To confirm the repeatability, six replicates of the same sample were extracted and analyzed. Variations were expressed in terms of relative standard deviation (RSD) in all the tests.

## 3. Results and Discussion

### 3.1. Optimization of Extraction Procedure

In order to obtain satisfactory extraction efficiency, several extraction solvents including water, 50% methanol (v/v), and methanol were examined. The 50% methanol (v/v) solvent was chosen and used as the extraction solvent due to its high yield of target compounds. Reflux and ultrasonic extraction methods were similarly effective in the extraction of the target analytes. The ultrasonic extraction method was ultimately chosen because of its flexibility. Different extraction times (30, 45, and 60 min) were compared, which showed similar percent yields and as such 30 minutes was chosen as the ideal extraction time.

### 3.2. Optimization of LC-QQQ-MS Chromatographic Conditions

Chromatographic conditions of the mobile phase and gradient elution system were optimized in this study. In order to achieve good resolution and symmetric peak shapes of the nine reference compounds, we chose methanol-water (with and without acid) and acetonitrile-water (with and without acid) to optimize the mobile phase. We also optimized the column temperature from 25 to 45°C with 5°C in one step. Finally, acetonitrile-water (with 0.1% formic acid) and column temperature of 35°C were chosen which showed good resolution of adjacent peaks within a short time.

### 3.3. UHPLC-Q-TOF-MS Fingerprint Analysis

**Figure 2:**
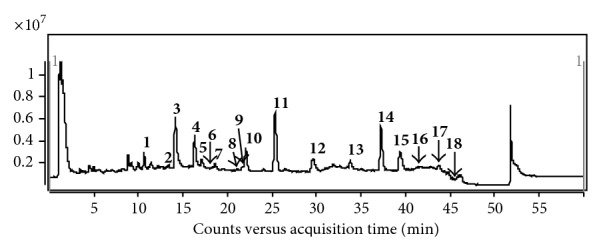
UHPLC-Q-TOF fingerprint of extract of* Eclipta prostrata *L.

Figure 2 shows the total ion current (TIC) chromatogram of the extract from* E. prostrata *(S9). A total of 18 compounds were identified and 13 of them confirmed by comparing their MS features and retention times with those of reference compounds. According to the structural characteristics, the 18 compounds can be grouped into three types, namely, flavonoids, triterpenoids, and other types.

#### 3.3.1. Identification of Flavonoids

**Figure 3:**
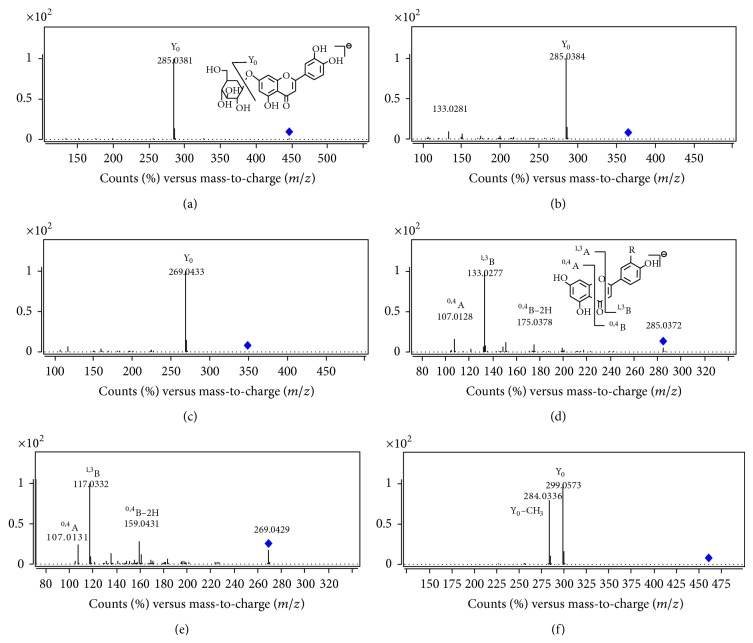
MS/MS spectra of flavonoids. ((a) compound** 1**; (b) compound** 3**; (c) compound** 4**; (d) compound** 5**; (e) compound** 8**; and (f) compound** 7**).

Seven flavonoids including two isoflavonoids were unambiguously or tentatively identified according to the UV spectra and MS fragmentation pathway. Compounds** 1** and** 7 **showed [M–H]^−^ at* m/z *447.0949 and 461.1094, respectively (Table 2). Similar diagnostic fragment Y_0_ ions were observed in their MS/MS spectra (Figure 3), suggesting these compounds are* O*-glycosyl flavonoids. Based on the UV, MS fragmentation pathway, and retention times, compounds** 1** and** 7** were identified as luteolin-7-*O*-*β*-D-glucoside and 7-*O*-methylorobol-4′-*O*-*β*-D-glucoside, respectively.

Compounds** 3** and** 4** produced [M–H]^−^ at* m/z *364.9990 and 349.0034 which correspond to C_15_H_10_O_9_S and C_15_H_10_O_8_S, respectively. In MS/MS spectra, Y_0_ ions at* m/z *285.0385 and 269.0434 were obtained. According to UV, MS, and literature [15],** 3** and** 4 **were tentatively identified as luteolin sulfate and apigenin sulfate.

Three flavone aglycones were identified corresponding to the diagnostic fragment ions ^1,3^B, ^0,4^A, and ^0,4^B-2H. Finally, Compounds** 5**,** 8**, and** 9** were unambiguously identified as luteolin, apigenin, and 3′-hydroxybiochanin A by comparing their retention times, MS/MS fragment data, and UV spectra with reference compounds.

#### 3.3.2. Identification of Triterpenoids

**Figure 4:**
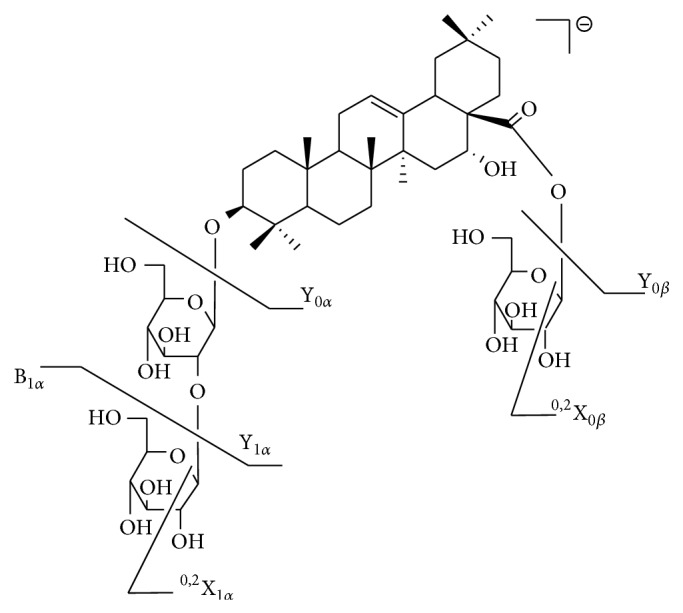
The fragmentation nomenclature of compound** 10**.

The triterpenoids were the major components isolated from* E. prostrata* and compounds** 10**–**16** were identified as triterpenoid saponins. In order to assist in structural elucidation, compound** 10 **was chosen as an example to elucidate the nomenclature (Figure 4). The ions retaining the charges on the main core structures were termed Y representing glycosidic cleavages and X for cross-ring cleavages [16]. Cross ring cleavage ions were designated by superscript numbers indicating cleavage of the two bonds. The oligosaccharide chain at C-3 was defined as the*α*-chain whereas the one at C-28 was the  *β*-chain.

**Figure 5:**
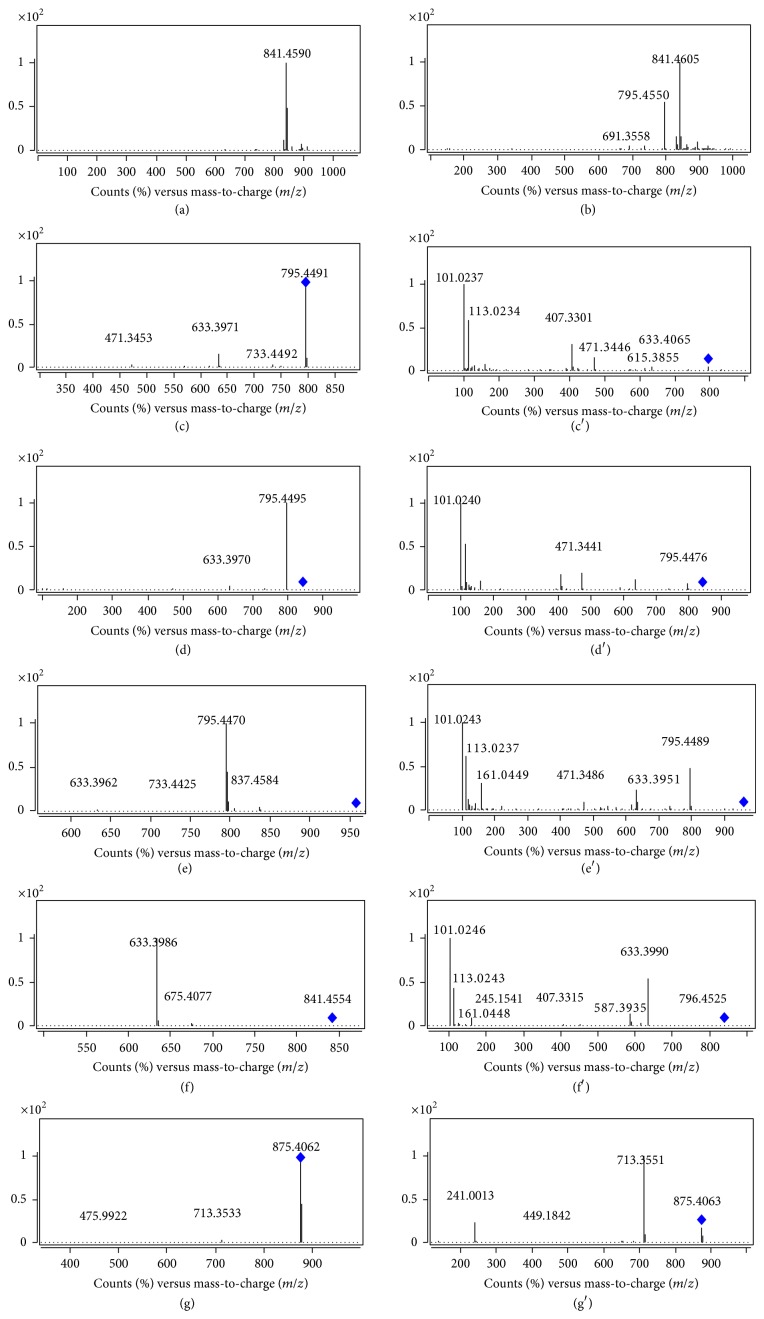
MS and MS/MS spectra of compounds** 10**,** 11, **and** 13**. ((a) MS spectrum of** 11**; (b) MS spectrum of** 13**; (c) MS/MS spectrum of [M–H]^−^ of** 13**, CE 35 V; (c′) MS/MS spectrum of [M–H]^−^ of** 13**, CE 70 V; (d) MS/MS spectrum of [M+HCOO]^−^ of** 13**, CE 35 V; (d′) MS/MS spectrum of [M+HCOO]^−^ of** 13**, CE 70 V; (e) MS/MS spectrum of [M–H]^−^ of** 10**, CE 35 V; (e′) MS/MS spectrum of [M–H]^−^ of** 10**, CE 70 V; (f) MS/MS spectrum of [M+HCOO]^−^ of** 11**, CE 35 V; (f′) MS/MS spectrum of [M+HCOO]^−^ of** 11**, CE 70 V; (g) MS/MS spectrum of [M–H]^−^ of** 12**, CE 35 V; (g′) MS/MS spectrum of [M–H]^−^ of** 12**, CE 70 V).

**Figure 6:**
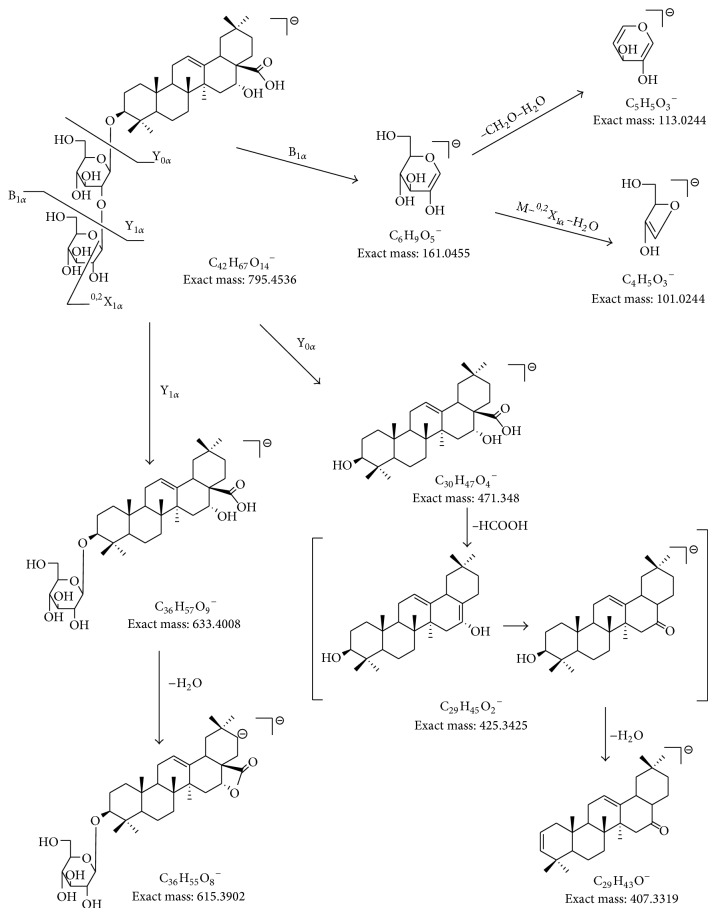
Fragmentation pattern of compound** 13**.

Since the molecular masses of saponins were usually large, we used two collision energies (CE) to elucidate the structures. Compound** 13** showed* m/z *795.4550 [M–H]^−^ and 841.4605 [M+HCOO]^−^ which correspond to C_42_H_68_O_14_ in MS spectrum (Figure 5(b)). As shown in Figures 5 (c, c′, d, d′), the MS/MS spectra of [M–H]^−^ and [M+HCOO]^−^ of** 13** were significantly different due to different CE. A collision energy of 35 V produced a base peak of [M–H]^−^ (*m/z *795.4491) whereas that of 70 V produced M-^0,2^X_1*α*_-H_2_O (*m/z *101.0237). As shown in Figure 5(c′), diagnostic fragment ions such as Y_0*α*_, Y_1*α*_, Y_1*α*_-H_2_O, Y_0*α*_-HCOOH, Y_0*α*_-HCOOH-H_2_O, B_1*α*_, B_1*α*_-CH_2_O-H_2_O, and M-^0,2^X_1*α*_-H_2_O were clearly observed.  Figure 6 shows the elucidation of the detailed fragment cleavage pathway and similar fragmentation pathways were observed in the MS/MS spectrum of [M+HCOO]^−^ (Figure 5(d′)). Through UV, MS fragmentation pathway, and retention times, compound** 13 **was unambiguously identified as eclalbasaponin IV.

Compound** 11** was the isomer of** 13**. However only [M+HCOO]^−^ ion (*m/z* 841.4590) was observed in its MS spectrum (Figure 5(a)). In the MS/MS spectra (CE 70 V), similar diagnostic fragment ions (Y_0*β*_, Y_0*β*_-HCOOH, Y_0*α*_-HCOOH-H_2_O, B_0*α*_, B_0*α*_-CH_2_O-H_2_O, and M-^0,2^X_0*α*_-H_2_O) were observed. Compound** 11** was subsequently identified as ecliptasaponin C.

Compound** 10** produced [M–H]^−^ and [M+HCOO]^−^ ions at* m/z* 957.5055 and 1003.5110 (corresponding to C_48_H_78_O_19_), which were 162 Da higher than that of compound** 13**. From the MS/MS fragment ions, UV spectrum, and literatures [9, 15], compound** 10 **was tentatively identified as eclalbasaponin III. Based on the UV, MS fragmentation pathway, and retention times, compounds** 14** and** 16** were unambiguously identified as isomers, namely, eclalbasaponin A and echinocystic acid 28-*O*-*β*-D-glucopyranoside, respectively.

Compound** 15** showed [M–H]^−^ ion at* m/z* 713.3582 (corresponding to C_36_H_58_O_12_S) which is 80 Da higher than that of** 14** (*m/z* 633.4023). From the MS spectrum, we could deduce that compound** 15** was the sulphate of compound** 14**. An* m/z* of 241.0007 was a significant diagnostic ion which indicated the location of SO_3_ on the glucose*α*-chain. In the MS/MS spectra of [M–H]^−^ (CE 70 V), diagnostic fragment ions, such as M-H-CO-2H, M-H-H_2_O-CO_2_, Y_0*α*_, Y_0*α*_-HCOOH-H_2_O, B_0*α*_, B_0*α*_-CH_2_O-H_2_O, and M-^0,2^X_0*α*_-H_2_O, were observed. According to the UV, MS fragmentation pathway, and literature [7], compound** 15** wasidentified as eclalbasaponin V tentatively and compound** 12** was 162 Da higher than** 15**. From the MS/MS fragmentation pathway (Table 2 and Figure 5(g′)), the 162 Da could be deduced as a  *β*-chain linked glucose. Finally, compound** 12** was tentatively identified as eclalbasaponin VI [15].

Two aglycones were also identified according to their UV, MS fragmentation pathway, and retention times. Compounds** 17** and** 18** were identified as echinocystic acid and 3-oxo-16*α*-hydroxy-olean-12-en-28-oic acid, respectively.

#### 3.3.3. Identification of Other Compounds

Two compounds (**2** and** 6**) were unambiguously identified as 3,4-dihydroxy-benzoic acid ethyl ester and wedelolactone, respectively, by comparing their UV, exact molecular masses, MS/MS spectra (Table 2), and retention times.

### 3.4. Validation of Quantitative Method

#### 3.4.1. Linearity

A series of standard solutions with seven different concentrations were analyzed by an established method in triplicate. Every calibration curve was plotted based on linear regression analysis of the integrated peak areas (*Y*) versus concentrations (*X*, *μ*g/mL) as listed in Table 3. Calibration curves were linear with correlation coefficients (*R* ^2^) above 0.9990 for all analytes.

#### 3.4.2. LOD and LOQ

The stock solutions containing nine reference compounds were diluted to a series of appropriate concentrations, using 50% methanol (v/v), and injected into LC/MS for analysis. The LOD and LOQ under the chromatographic conditions were determined at approximate signal-noise (*S*/*N*) ratios of 3 and 10, respectively. The results were given in Table 3.

#### 3.4.3. Precision and Repeatability

Intra- and interday precisions were performed by repetitive injections on the same day (intraday) for a total of six injections and on three consecutive days (interday). RSD values for both intra- and interday precision were below 2.5% (Table 4).

The analytic repeatability was examined by the injection of six different samples (S9), which were prepared with the same sample preparation procedure. The repeatability of the solution was less than 2.99% (Table 4).

#### 3.4.4. Accuracy

The accuracy of the method was determined by spiking an appropriate amount of each crude* E. prostrata* extract sample (S9) with accurate amounts of the nine reference standards and each sample was analyzed in triplicate. The results showed good accuracy with average recovery from 95.41% to 104.76% for the compounds concerned with RSD < 4.20% (Table 4).

These results show that the LC/MS method was precise, accurate, and sensitive enough for simultaneously quantitative evaluation of the nine compounds from the aerial part of* E. prostrata*.

### 3.5. Application of Quantitative Method

**Figure 7:**
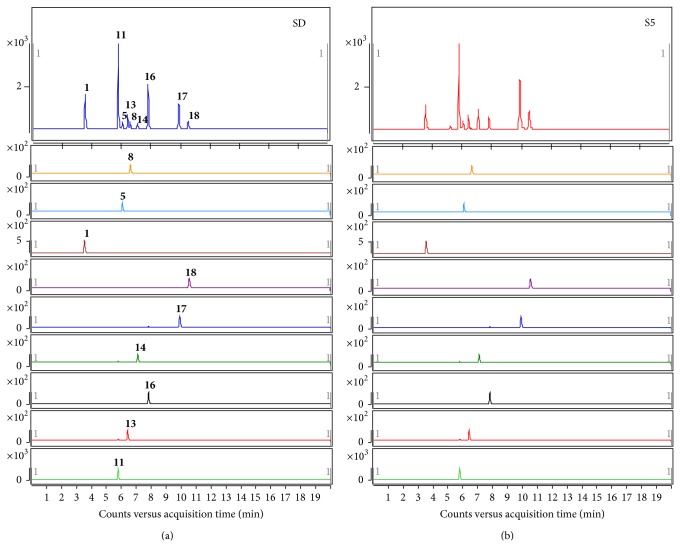
Typical MRM chromatograms of mix standard (a) and sample (b).

The method can be used for simultaneous analysis of thirteen* E. prostrata* samples from different habitats of China (S1: Hunan; S2: Hebei; S3: Henan; S4: Henan; S5: Hebei; S6: unknown; S7: Jiangsu; S8: unknown; S9: Hebei; S10: unknown; S11: unknown; S12: Hebei; and S13: Anhui) (Figure 7). The saponins (**11**,** 13**,** 14**, and** 16**) were the major constituents among the nine compounds analysed and they showed relatively high variations (Table 5). Compound** 11** was the most abundant among the nine compounds and was also recording high variations (426.16–13056.45 *μ*g/g). This high degree of contents variability among the thirteen samples from different geographical locations could be due to various factors such as geographical source, climate, harvest time, and storage condition.

## 4. Conclusion

The two different LC/MS methods of analyses could be used for the rapid profiling and determination of major constituents from* E. prostrata*. The qualitative and quantitative methods could also be reliable tools for quality control analyses.

## Figures and Tables

**Table 1:** MRM parameters for quantitative analysis.

Compounds	Ion pairs (*m*/*z*)	Fragmentor (V)	CE (V)	Dwell time (ms)
**1**	447.1 → 285.0	240	26	100
**11**	841.5 → 633.4	210	30	100
**5**	285.0 → 133.1	170	30	100
**13**	795.5 → 633.4	310	40	100
**8**	269.0 → 117.1	150	34	100
**14**	633.4 → 587.4	270	38	100
**16**	679.4 → 471.3	150	22	100
**17**	471.3 → 407.3	230	38	100
**18**	469.3 → 407.3	220	34	100

**Table 2:** Characterization of compounds from *E. prostrata* by UHPLC-DAD-Q-TOF.

Number	RT (min)	UV *λ*_max⁡_ (nm)	Formula	*m*/*z* (experimental)	Error (ppm)	Product ions (*m*/*z*, relative abundance, diagnostic ions)	Structural elucidation
**1**	10.93	228, 254, 350	C_21_H_20_O_11_	447.0949 [M–H]^−^	−3.66	285.0381 (100) [Y_0_]	Luteolin-7-*O*-*β*-D-glucoside

**2**	14.13	230, 289, 330	C_9_H_10_O_4_	181.0514 [M–H]^−^	−4.30	108.0256 (100) [M-CO_2_-C_2_H_5_]	3,4-Dihydroxy-benzoic acid ethyl ester

**3**	14.53	226, 254, 350	C_15_H_10_O_9_S	364.9990 [M–H]^−^	−4.80	285.0385 (100) [Y_0_]	Luteolin sulfate

**4**	16.70	228, 268, 340	C_15_H_10_O_8_S	349.0034 [M–H]^−^	−3.08	269.0434 (100) [Y_0_]	Apigenin sulfate

**5**	17.45	246, 350	C_15_H_10_O_6_	285.0409 [M–H]^−^	−1.48	175.0378 (10) [^0,4^B-2H]	Luteolin
						133.0277 (100) [^1,3^B]	
						107.0128 (20) [^0,4^A]	

**6**	18.31	228, 260	C_16_H_10_O_7_	313.0363 [M–H]^−^	−3.04	298.0112 (100) [M-CH_3_]	Wedelolactone
						270.0158 (20) [M-CH_3_-CO]	
						242.0212 (10) [M-CH_3_-2CO]	

**7**	18.81	245	C_22_H_22_O_11_	461.1094 [M–H]^−^	−0.92	299.0573 (100) [Y_0_]	7-*O*-Methylorobol-4′-*O*-*β*-D-glucoside
						284.0336 (80) [Y_0_-CH_3_]	

**8**	21.61	228, 267, 340	C_15_H_10_O_5_	269.0451 [M–H]^−^	1.69	159.0431 (30) [^0,4^B-2H]	Apigenin
						117.0332 (100) [^1,3^B] 107.0131 (24) [^0,4^A]	

**9**	22.32	270, 328, 350	C_16_H_12_O_6_	299.0556 [M–H]^−^	1.77	284.0330 (100) [M-CH_3_]	3′-Hydroxybiochanin A
						256.0372 (40) [M-CH_3_-CO]	
						255.0303 (50) [M-CH_2_-CO-H_2_]	
						239.0350 (18) [M-CH_4_O-CO]	
						227.0353 (67) [M-CH_2_-2CO-H_2_]	
						211.0402 (29) [M-CH_4_O-2CO]	

**10**	22.32	210	C_48_H_78_O_19_	957.5055 [M–H]^−^ (CE 35)	0.91	837.4584 (4) [ ^0,2^X_0*β*_]	Eclalbasaponin III
						795.4470 (100) [Y_0*β*_]	
						733.4425 (1) [Y_0*β*_-H_2_O-CO_2_]	
						633.3962 (1) [Y_0*β*_-glc]	
				957.5055 [M–H]^−^ (CE 70)	0.91	795.4489 (50) [Y_0*β*_]	
						733.4421 (4) [Y_0*β*_-H_2_O-CO_2_]	
						633.3951 (22) [Y_0*β*_-glc]	
						471.3486 (10) [Y_0*β*_-2glc]	
						161.0449 (31) [B_1*α*_]	
						113.0237 (62) [B_1*α*_-CH_2_O-H_2_O]	
						101.0243 (100) [M-^0,2^X_1*α*_-H_2_O]	
				1003.5110 [M + HCOO]^−^ (CE 35)	1.10	837.4583 (6) [ ^0,2^X_0*β*_]	
						795.4481 (100) [Y_0*β*_]	
						733.4424 (1) [Y_0*β*_-H_2_O-CO_2_]	
						633.3934 (1) [Y_0*β*_-glc]	
				1003.5110 [M + HCOO]^−^ (CE 70)	1.10	795.4496 (100) [Y_0*β*_]	
						733.4477 (8) [Y_0*β*_-H_2_O-CO_2_]	
						633.3968 (32) [Y_0*β*_-glc]	
						615.3851 (6) [Y_0*β*_-glc-H_2_O]	
						571.3985 (5) [Y_0*β*_-glc-H_2_O-CO_2_]	
						471.3447 (10) [Y_0*β*_-2glc]	
						161.0444 (36) [B_1*α*_]	
						113.0239 (56) [B_1*α*_-CH_2_O-H_2_O]	
						101.0240 (89) [M- ^0,2^X_1*α*_-H_2_O]	

**11**	25.75	210	C_42_H_68_O_14_	841.4590 [M + HCOO]^−^ (CE 35)	0.19	675.4077 (4) [ ^0,2^X_0*β*_]	Ecliptasaponin C
						633.3986 (100) [Y_0*β*_]	
				841.4590 [M + HCOO]^−^ (CE 70)	0.19	633.3990 (54) [Y_0*β*_]	
						587.3935 (13) [Y_0*β*_-HCOOH]	
						407.3315 (1) [Y_0*α*_-HCOOH-H_2_O]	
						161.0448 (10) [B_0*α*_]	
						113.0243 (43) [B_0*α*_-CH_2_O-H_2_O]	
						101.0246 (100) [M- ^0,2^X_0*α*_-H_2_O]	

**12**	30.27	210	C_42_H_68_O_17_S	875.4116 [M–H]^−^ (CE 35)	−1.28	875.4062 (100) [M–H]	Eclalbasaponin VI
						713.3533 (3) [Y_0*β*_]	
				875.4116 [M–H]^−^ (CE 70)	−1.28	875.4063 (16) [M–H]	
						713.3551 (100) [Y_0*β*_]	
						651.3543 (2) [Y_0*β*_-H_2_O-CO_2_]	
						241.0013 (21) [B_0*α*_]	

**13**	34.04	210	C_42_H_68_O_14_	795.4550 [M–H]^−^ (CE 35)	−1.77	795.4491 (100) [M–H]	Eclalbasaponin IV
						733.4492 (4) [M–H-H_2_O-CO_2_]	
						633.3971 (16) [Y_1*α*_]	
						471.3453 (3) [Y_0*α*_]	
				795.4550 [M–H]^−^ (CE 70)	−1.77	795.4509 (5) [M–H]	
						633.4065 (4) [Y_1*α*_]	
						615.3855 (3) [Y_1*α*_-H_2_O]	
						471.3446 (16) [Y_0*α*_]	
						407.3301 (31) [Y_0*α*_-HCOOH-H_2_O]	
						161.0431 (10) [B_1*α*_]	
						113.0234 (57) [B_1*α*_-CH_2_O-H_2_O]	
						101.0237 (100) [M- ^0,2^X_1*α*_-H_2_O]	
				841.4605 [M + HCOO]^−^ (CE 35)	−1.79	795.4495 (100) [M–H]	
						733.4480 (2) [M–H-H_2_O-CO_2_]	
						633.3971 (5) [Y_1*α*_]	
						471.3432 (1) [Y_0*α*_]	
				841.4605 [M + HCOO]^−^ (CE 70)	−1.79	795.4476 (7) [M–H]	
						633.3989 (12) [Y_1*α*_]	
						615.3834 (2) [Y_1*α*_-H_2_O]	
						471.3439 (20) [Y_0*α*_]	
						407.3295 (18) [Y_0*α*_-HCOOH-H_2_O]	
						161.0450 (11) [B_1*α*_]	
						113.0236 (54) [B_1*α*_-CH_2_O-H_2_O]	
						101.0241 (100) [M- ^0,2^X_1*α*_-H_2_O]	

**14**	37.60	210	C_36_H_58_O_9_	633.4023 [M–H]^−^ (CE 35)	−0.19	633.3985 (100) [M–H]	Eclalbasaponin A
						587.3922 (4) [M–H-HCOOH]	
						571.3966 (1) [M–H-H_2_O-CO_2_]	
				633.4023 [M–H]^−^ (CE 70)	−0.19	587.3916 (3) [M–H-HCOOH]	
						585.3706 (5) [M–H-HCOOH-H_2_]	
						407.3281 (12) [Y_0*α*_-HCOOH-H_2_O]	
						161.0434 (1) [B_0*α*_]	
						113.0235 (32) [B_0*α*_-CH_2_O-H_2_O]	
						101.0241 (100) [M- ^0,2^X_0*α*_-H_2_O]	

**15**	36.79	210	C_36_H_58_O_12_S	713.3582 [M–H]^−^ (CE 35)	−0.77	713.3545 (100) [M–H]	Eclalbasaponin V
				713.3582 [M–H]^−^ (CE 70)	−0.77	713.3544 (62) [M–H]	
						683.3073 (17) [M–H-CO-2H]	
						651.3541 (10) [M–H-H_2_O-CO_2_]	
						471.3464 (1) [Y_0*α*_]	
						407.3306 (3) [Y_0*α*_-HCOOH-H_2_O]	
						241.0007 (100) [B_0*α*_]	
						113.0234 (7) [B_0*α*_-CH_2_O-H_2_O]	
						101.0241 (10) [M- ^0,2^X_0*α*_-H_2_O]	

**16**	41.86	210	C_36_H_58_O_9_	679.4053 [M + HCOO]^−^ (CE 35)	1.59	471.3430 (100) [Y_0*α*_]	Echinocystic acid 28-*O*-*β*-D-glucopyranoside
						407.3280 (3) [Y_0*α*_-HCOOH-H_2_O]	
				679.4053 [M + HCOO]^−^ (CE 70)	1.59	471.3393 (18) [Y_0*α*_]	
						425.3376 (11) [Y_0*α*_-HCOOH]	
						407.3301 (100) [Y_0*α*_-HCOOH-H_2_O]	
						391.2945 (12) [Y_0*α*_-CO_2_-2H_2_O]	

**17**	44.61	210	C_30_H_48_O_4_	471.3470 [M–H]^−^ (CE 35)	2.06	471.3439 (84) [M–H]	Echinocystic acid
						407.3281 (100) [M–H-HCOOH-H_2_O]	
						425.3407 (10) [M–H-HCOOH]	

**18**	44.75	210	C_30_H_46_O_4_	469.3311 [M–H]^−^ (CE 35)	2.51	469.3283 (9) [M–H]	3-Oxo-16*α*-hydroxy-olean-12-en-28-oic acid
						423.3222 (1) [M–H-HCOOH]	
						407.3296 (100) [M–H-HCOOH-H_2_O]	

**Table 3:** Calibration curves, LOD, and LOQ of nine compounds.

Compounds	Calibration curves	*R* ^2^	Linear range (μg/mL)	LOD (ng/mL)	LOQ (ng/mL)
**1**	*Y* = 8272*X* + 203	0.9996	0.028–20.17	3.37	11.22
**11**	*Y* = 6347.7*X* + 4182.9	0.9992	0.070–52.28	1.10	3.68
**5**	*Y* = 3350*X* + 4.63	0.9998	0.012–8.59	1.77	5.90
**13**	*Y* = 1190*X* + 462.4	0.9993	0.060–43.89	4.41	14.71
**8**	*Y* = 5271*X* + 245.1	0.9996	0.008–5.78	1.94	6.47
**14**	*Y* = 822.7*X* + 261.7	0.9990	0.033–23.89	2.24	7.47
**16**	*Y* = 27335.1*X* − 318.5	0.9996	0.013–9.63	1.36	4.54
**17**	*Y* = 6640.4*X* + 865.8	0.9994	0.013–9.39	2.00	6.69
**18**	*Y* = 2577.4*X* + 393.6	0.9991	0.010–7.13	2.47	8.23

**Table 4:** Precision, recovery, and repeatability of nine compounds.

Compounds	Precision (RSD, %, *n* = 6)		Recovery (%, *n* = 6)		Repeatability (%) (RSD, *n* = 6)
	Intraday	Interday	Mean	RSD	
**1**	1.00	1.16	97.21	2.39	1.75
**11**	0.66	1.98	95.41	2.63	1.12
**5**	1.56	1.60	97.82	3.99	2.99
**13**	0.97	2.49	101.28	3.84	2.35
**8**	1.83	2.50	96.00	3.88	2.45
**14**	2.37	2.46	103.25	2.85	1.99
**16**	1.64	2.13	104.76	3.49	2.44
**17**	0.57	2.45	96.44	2.37	2.95
**18**	1.64	2.42	103.56	4.20	2.64

**Table 5:** Average contents of nine compounds in *E. prostrata* (mean ± SD, *μ*g/g, *n* = 3).

	**1**	**11**	**5**	**13**	**8**	**14**	**16**	**17**	**18**
S1	12.15 ± 0.06	8827.39 ± 29.38	7.29 ± 0.09	473.81 ± 4.18	ND	1438.43 ± 6.66	342.60 ± 3.45	342.60 ± 3.33	354.75 ± 3.84
S2	724.00 ± 10.42	13056.45 ± 20.30	154.09 ± 1.91	1369.73 ± 5.54	ND	3032.97 ± 8.80	286.18 ± 3.34	454.95 ± 4.56	369.34 ± 4.96
S3	2237.32 ± 14.13	3128.35 ± 15.19	350.57 ± 0.98	326.22 ± 3.23	8.82 ± 0.06	1188.04 ± 2.15	12.17 ± 0.09	17.04 ± 0.09	ND
S4	1198.61 ± 10.25	2674.59 ± 12.14	210.50 ± 1.23	304.61 ± 2.02	ND	973.25 ± 2.23	64.39 ± 1.11	173.35 ± 1.10	118.87 ± 2.83
S5	104.063 ± 1.21	426.16 ± 8.23	74.33 ± 0.46	411.30 ± 2.01	ND	1194.25 ± 4.56	14.87 ± 0.10	374.13 ± 3.23	421.21 ± 3.75
S6	921.77 ± 6.48	2092.68 ± 13.15	201.79 ± 1.56	465.87 ± 4.09	ND	1542.10 ± 5.14	24.91 ± 0.07	146.99 ± 1.18	89.69 ± 1.56
S7	27.241 ± 0.25	5757.80 ± 15.20	44.58 ± 0.20	928.68 ± 6.28	ND	2439.33 ± 5.85	136.21 ± 1.89	307.08 ± 3.33	222.88 ± 2.34
S8	392.41 ± 0.86	10565.27 ± 40.59	193.72 ± 2.02	1204.55 ± 7.93	ND	2799.03 ± 7.19	139.08 ± 1.93	317.90 ± 3.44	206.14 ± 3.32
S9	3748.13 ± 18.18	4099.054 ± 10.12	428.07 ± 2.88	380.79 ± 2.89	14.37 ± 0.10	1057.74 ± 6.34	17.42 ± 0.09	69.69 ± 0.69	7.47 ± 0.06
S10	665.41 ± 2.23	3160.69 ± 10.89	151.46 ± 1.45	322.77 ± 2.26	ND	1104.88 ± 5.25	52.14 ± 0.21	216.01 ± 3.74	136.56 ± 1.54
S11	556.11 ± 4.56	8980.05 ± 13.15	226.93 ± 2.77	1017.46 ± 4.87	ND	2496.26 ± 8.29	127.18 ± 1.10	192.02 ± 3.21	137.18 ± 1.34
S12	698.32 ± 7.19	8260.18 ± 20.86	177.08 ± 1.23	905.33 ± 4.98	ND	2239.63 ± 9.10	67.34 ± 0.42	207.00 ± 2.34	114.73 ± 1.09
S13	531.12 ± 4.42	7837.12 ± 19.38	269.30 ± 2.56	1269.20 ± 6.30	33.19 ± 0.22	2822.66 ± 14.14	119.69 ± 1.85	321.66 ± 5.02	182.03 ± 1.21

ND: not detected; S1: Hunan; S2: Hebei; S3: Henan; S4: Henan; S5: Hebei; S6: unknown; S7: Jiangsu; S8: unknown; S9: Hebei; S10: unknown; S11: unknown; S12: Hebei; S13: Anhui.
